# Real-World Outcomes of Brexanolone to Treat Postpartum Depression

**DOI:** 10.1177/26884844251383702

**Published:** 2025-10-06

**Authors:** Melanie Barrett, Jennifer Hettema, Constance Youngman, Samuel T. Wilkinson, Rachel Dalthorp

**Affiliations:** ^1^ LifeStance Health, Scottsdale, Arizona, USA.; ^2^ Department of Psychiatry, School of Medicine, Yale University, New Haven, Connecticut, USA.

**Keywords:** brexanolone, zuranolone, postpartum depression, postpartum anxiety, reproductive psychiatry, perinatal mental health

## Abstract

**Background:**

While the efficacy of brexanolone, a U.S. Food and Drug Administration-approved infusion treatment for postpartum depression (PPD), has been demonstrated in controlled trials, few reports are available describing its real-world effectiveness.

**Methods:**

Leveraging real-world clinical data, the current report describes the characteristics and treatment outcomes of a sample of women (*N* = 150) receiving brexanolone treatment for PPD in a residential-style outpatient treatment center with up to 12 months of follow-up. The sample had a mean age of 30.0 years (SD = 4.5), with almost one-third (31.3%) on hormonal birth control, and most (91.3%) were taking concomitant psychiatric medications.

**Results:**

Participants reported significant decreases in depression and anxiety symptoms from pretreatment to posttreatment that were sustained across a 12-month follow-up. Edinburgh postnatal depression scale (EPDS) scores decreased from pretreatment (*M* = 19.9, SD = 4.1) to posttreatment (*M* = 10.5, SD = 4.5, *p* < 0.001, within-group effect size [Cohen’s d] of 2.2). At 12 months follow-up, EPDS scores remained significantly lower among the retained subset of patients (*N* = 64, *M* = 9.2, SD = 6.5). Among a subset who underwent formal assessments for anxiety, scores on the perinatal anxiety screening scale decreased from pretreatment (*M* = 60.1, SD = 17.1) to posttreatment (*M* = 33.3, SD = 22.1, *p* < 0.01, within-group effect size of 1.4). Response and remission rates for depression were 68.7% and 46.7%, respectively, posttreatment, and 73.4% and 60.9%, respectively, at 12-month follow-up. Importantly, 15 participants had a diagnosis of bipolar depression and were prescribed an antipsychotic or mood stabilizer at the time of treatment, there were no episodes of mania or hypomania reported during the follow-up period.

**Conclusions:**

Given the shared mechanism of action between brexanolone and the recently approved oral medication for PPD, zuranolone, the applicability of findings and relevance to the more accessible oral drug, zuranolone, are explored.

## Introduction

The transition to motherhood can significantly impact a woman’s physical, mental, and emotional well-being and is a high-risk time for the onset or worsening of mental health disorders. Postpartum depression (PPD) is an episode of major depressive disorder emerging during pregnancy and up to 4 weeks postpartum, Diagnostic and Statistical Manual of Mental Disorders, Fifth Edition, (DSM-5) or 12 months postpartum, American College of Obstetricians and Gynecologists (ACOG), depending on the definition.^1,2^ The global prevalence of PPD among healthy mothers with no prior history of mental health disorders is 17%.^3^ In women with a history of childhood adversity, depression prior to pregnancy, or psycho-social stressors, prevalence is even higher.^4,5^ PPD impacts the mother, baby, and entire family, with the potential for long-term and generational consequences.^6^ Negative outcomes include maternal suicidal behavior, impaired mother–child interactions and bonding, and negative health, psychological, and cognitive outcomes for children.^7,8^ According to a recent study of 2017 births in the United States, the overall cost of perinatal mood and anxiety disorders was $14 billion, with each case costing approximately $81,000.^9^

PPD is underrecognized and undertreated, with as few as 5% of women treated to remission of symptoms.^10^ Left untreated, PPD can persist for years,^3^ making early and effective treatment critical.^11^ The American Academy of Pediatrics and the US Preventive Services Task Force both recommend frequent screening during pregnancy and postpartum to identify women with PPD.^12,13^

Research suggests that psychosocial and psychological interventions can be effective in reducing depressive symptomatology in PPD, though there is significant heterogeneity in effects, and symptom improvements often take months.^14,15^ Until recently, there were no U.S. Food and Drug Administration (FDA) approved treatments for PPD. Instead, the disorder has been pharmacologically treated in practice as a variant of major depression with antidepressants, particularly selective serotonin reuptake inhibitors (SSRIs), which show promising effects for some women, though results are mixed, a significant proportion of women do not respond, and treatment effects can be delayed^16,17^ Given the significant hormonal changes observed during the perinatal period and their known effects on mood, some preliminary work has also explored the role of reproductive hormones and metabolites, including progesterone, allopregnanolone, and estrogen on PPD with mixed results.^17^

Researchers leveraged basic science data regarding the pathophysiology of PPD, including data suggesting that allopregnanolone, a neuroactive steroid and metabolite of the reproductive steroid progesterone, falls precipitously following childbirth, initiating an episode of PPD for some women. Allopregnanolone is a potent allosteric modulator of gamma-aminobutyric acid (GABA-A) neurons implicated in mood regulation.^17^ and hypothalamus–pituitary–adrenal axis response to stress. In 2019, the FDA approved brexanolone (Brexanolone® [brexanolone] injection, Sage Therapeutics, Inc., Cambridge, MA), a neurosteroid that binds to and interacts with GABA-A synaptic and extrasynaptic receptors.^18^ It is administered intravenously *via* infusion, in a risk evaluation and mitigation strategy (REMS; a drug safety program that the FDA can require for specific medications) certified and monitored setting, across 60 hours.

Brexanolone was approved by the FDA following three double-blind randomized controlled trials. The first phase 2 trial comparing brexanolone to placebo included 21 inpatient women with severe PPD and found significantly larger reductions in depression scores posttreatment and up to one month follow-up among treated patients, similar rates of adverse events across groups, and no serious adverse events or discontinuations.^19^ Two follow-up, larger, phase 3 trials targeted women with severe depression (Hamilton depression rating scale [HAM-D] >26; *N* = 138; trial 1) or moderate depression (HAM-D = 20–25; *N* = 108; trial 2) and compared brexanolone 90 μg/kg per hour (BRX90), brexanolone 60 μg/kg/h (BRX60), or placebo (trial 1) or brexanolone 90 μg/kg/h (BRX90) or placebo (trial 2) at one month follow-up.^20^ These studies also demonstrated significant reductions in depression scores for treated patients compared with placebo, with few (<1%) serious adverse events and comparable rates of adverse events. In a *post hoc* analysis of these three trials, outcomes on the HAM-D anxiety/somatization subscales and clinical global impression of improvement also revealed improved anxiety/somatization and insomnia symptoms in patients given a 60-hour infusion of brexanolone 90 μg/kg/h in the first 30 days posttreatment.^11^ As part of the FDA approval for brexanolone, because of the risk for excessive sedation and sudden loss of consciousness during treatment with brexanolone, the REMS Program necessitated that brexanolone be administered only in a medically supervised healthcare setting that provides monitoring during the entirety of the treatment.

Since its FDA approval in 2019, there has been limited reporting on brexanolone treatment in real-world settings. In one recent report, 16 women were treated at a university-based treatment center.^21^ Treated women reported significant reductions in depression as measured by the HAM-D from pretreatment (*m* = 24, SD = 3) to posttreatment (*m* = 8, SD = 3). The study team gathered follow-up data ranging from 3 to 16 months posttreatment from 11 of these patients who provided self-reported data, indicating that most were in remission. In a follow-up report, this team shared data that they had treated a larger cohort of 60 women at their university-based treatment center.^22^

While the above studies provide encouraging evidence regarding the feasibility of treating postpartum women with brexanolone and their data that suggest real-world clinical effects on depression may mirror those found in tightly controlled clinical trials, additional data is needed to evaluate the feasibility, safety, and effectiveness of brexanolone in community treatment settings. Additional data is also needed to describe the durability of response and impact on symptoms of anxiety. In the current study, we seek to describe the largest sample of patients receiving brexanolone to date (*n* = 150). Our findings include novel data describing the characteristics of treatment-seeking women, including pretreatment severity, psychiatric medication use, and use of hormonal birth control, as well as an expanded understanding of the effectiveness of brexanolone to include symptoms of anxiety and depression at up to 12 months posttreatment.

## Materials and Methods

### Ethical considerations

After obtaining approval from the Brany Institutional Review Board (24-12-431), we reviewed data from patients who underwent brexanolone treatment at our community-based clinical site. Data were extracted from electronic health records as well as internal quality improvement sources and deidentified for analysis.

### Patient population

This data was obtained from human subjects who included 150 women diagnosed with PPD who received brexanolone treatment at a REMS-certified outpatient treatment center in Oklahoma between the periods of December 2019 and September 2023. The sample represents all women treated during this time period. Inclusion criteria were treatment with brexanolone during the study window. Patients were self-referred or referred *via* other community clinicians to the program. Clinical determination of appropriateness for brexanolone treatment included a review of contraindications, medical and psychiatric history, concomitant medications, no current or planned pregnancy, and diagnostic criteria for PPD, including standardized depression rating scale and clinical interview data to support: (1) moderate to severe PPD, (2) a major depressive episode that began no earlier than the third trimester of pregnancy, and no later than 4 weeks postpartum, and (3) within one year postpartum. Patients presenting with onset or exacerbation of PPD symptoms at greater than 12 months postdelivery were considered for treatment based on their personal course of illness, patient preference, and insurance limitations. Treatment was billed to patients’ insurance, apart from 11 patients who were provided treatment at no cost following insurance denials. As part of treatment, data were collected on depression severity, anxiety severity, and, when indicated, obsessive–compulsive disorder (OCD) severity (Yale–Brown obsessive–compulsive scale [Y-BOCS]), concomitant medications, method of birth control, breastfeeding status, and time between delivery and treatment.

### Brexanolone treatment protocol

As a part of standard treatment, and consistent with REMS dosing and administration requirements, patients were administered a continuous intravenous infusion of brexanolone over 60 hours in a residential style outpatient treatment center with continuous monitoring by a minimum of two nursing staff members (registered nurse and licensed practical nurse) with daily physician on-site visits and continuous access to an on-call physician who provided oversite for the entire duration of the treatment. Dosing could be modified/terminated at the discretion of the supervising physician, following REMS requirements and package insert guidance for adverse events monitoring of patients during treatment.

Our residential style, outpatient treatment center was designed to feel like a homelike environment, with access to a living room-like space with comfortable seating, television, and games, a kitchen with healthy meals and snack options, and an outdoor patio. After recognizing that separation from baby and partner was a significant barrier to accepting and receiving treatment, we implemented a non-restrictive visiting policy to support family connectedness. Baby and a support person were invited to stay, and each private patient treatment room was equipped with two twin beds and, if needed, a pack and play for the visiting infant. Consistent with REMS requirements, mothers receiving treatment were not allowed to ambulate with the baby or hold the baby while unattended. Women were treated in a group setting, with up to six women in the facility at a time, creating a therapeutic milieu, with psychoeducation, group, and art therapy options.

### Assessment of patient characteristics

To describe the sample, multiple data elements were extracted from the electronic health record. These included patient age, time between delivery and treatment, presence and type of hormonal birth control, and current psychiatric medications, if any.

### Monitoring and assessment of safety

Patients were continuously monitored for adverse events for the entire duration of treatment. Consistent with the FDA REMS, patients were continuously monitored by pulse oximetry with an alarm during the infusion. Patients’ level of consciousness was assessed and documented every 2 hours during planned non-sleep periods during the infusion, utilizing the Glasgow Coma Scale. In addition, patients’ blood pressure, heart rate, intravenous patency, as well as verification of dose and rate, were obtained and documented throughout the infusion. Post-infusion forms were submitted following treatment per REMS requirements.

### Assessment of outcomes

As part of standard clinical care and quality improvement efforts, participants were administered standardized measures of depression, anxiety, and OCD across varying durations, aligning with pretreatment, posttreatment, 1-week, 4-week, 6-month, and 12-month clinical visits. Symptom rating tools included the following:

- Edinburgh postnatal depression scale (EPDS)^23^: The EPDS is a 10-question screening tool used to identify PPD.
- HAM-D^24^: The HAM-D is a 21-item scale that measures depression. It can be used before, during, and after treatment to monitor response to treatment.
- Generalized anxiety disorder-7 (GAD-7)^25,26^: The GAD-7 is a seven-item scale used to assess symptoms of Generalized Anxiety Disorder, identify symptoms, and assess their severity.
- Perinatal anxiety screening scale (PASS)^27,28^: The PASS is a 31-item self-report questionnaire used by clinicians to screen for anxiety amongst pregnant and postpartum patients.
- Y-BOCS^29,30^: The Y-BOCS is used to test and rate the severity of OCD. Within the naturalistic study sample, subsets of patients received specific rating tools for different durations based on changing clinical care approaches and instrument administration feasibility. All tools were universally administered, except for Y-BOCS, which was only administered to patients who reported anxiety that included intrusive or obsessive thoughts.

### Analyses

We conducted statistical analyses using IBM SPSS software, version 30 (IBM Corp., Armonk, NY, USA). We used descriptive statistics, including rates or proportions for categorical variables and means and standard deviations for continuous variables, to describe the size of the sample, rates of follow-up retention, characteristics of the patient population, and adverse events. To assess potential bias due to loss to follow-up, rates of severity at pretreatment were compared between those retained to those who were lost at the longest follow-up point using independent sample *t*-tests for all outcome measures (Table 2).

Effectiveness, as measured by within-group changes across time, was assessed using several strategies. Changes on the EPDS, HAM-D, GAD-7, PASS, and Y-BOCS from pretreatment to each follow-up point were used to calculate Cohen’s d effect sizes comparing pretreatment scores on each measure with each of the follow-up points. Cohen’s d effect sizes can be interpreted as small (*d* = 0.02), medium (*d* = 0.05), or large (*d* ≥ 0.08).^31,32^ Additionally, proportions of patients achieving remission and response were calculated for all measures at all available follow-up time points by calculating the percent of patients reporting symptoms that fell below the cutoff score for likely disorder within each measure (remission) or a reduction greater than or equal to 50% (or 35% for Y-BOCS) of the pretreatment score (response). Although this real-world effectiveness study was not powered for significance testing, we also conducted an exploratory one-way repeated measures analysis of variance (ANOVA). The follow-up period selected for inclusion in ANOVA testing was the longest measurement period with at least 50% follow-up retention. The assumption of sphericity was tested for each analysis using Machly’s test of sphericity and, in the case of significant *p* values (*p* > 0.05), degrees of freedom were appropriately adjusted. A *p* value of <0.05 was considered statistically significant.

## Results

### Patient characteristics

Table 1 describes the characteristics of the treatment sample. Women were, on average, 30 years old (SD = 4.5). Race and ethnicity data were not available. Time between delivery and treatment ranged from 20 to 421 days (*M* = 127, SD = 88). Most patients (80%) initiated treatment in the first 6 months postpartum, and nearly all (97%) initiated within the first 12 months postpartum. Four patients were seen in more than 12 months postpartum. Many patients reported that they were currently breastfeeding/pumping (42.0%) or weaned within the past four weeks (6.7%). A significant portion of women (31.3%) reported being on hormonal birth control at the time of treatment. Of these, 31 (20.7%) reported long-acting reversible contraceptives, and 16 (10.7%) reported use of oral contraceptives.

**Table 1.** Pretreatment Patient Characteristics

Characteristics	Result
Age in years (Mean, SD)	30.0, 4.5 *n* = 150
Days between delivery and treatment, mean (Mean, SD)	127.3, 88.0 *n* = 148
Lactation status (*n*, %)	Currently breastfeeding or pumping: 63/150 (42.0%) Weaned in last 4 weeks: 10/150 (6.7%)
Hormonal birth control (*n*, %)	Yes: 47/150 (31.3%) -LARC: 31 (20.7%) -OCP: 16 (10.7%)
Number of psychiatric medications (*n*, %)	None: 13/150 (8.7%) One: 90/150 (60.0%) Two: 39/150 (26.0%) Three: 5/150 (3.3%) Four or more: 3/150 (2.0%)
Number of patients taking at least one medication within each class (*n*, %)	SSRI: 114/150 (76.0%) SNRI: 4/150 (2.7%) Atypical antidepressant: 23/150 (15.3%) Atypical antipsychotic: 18/150 (12.0%) Mood stabilizer: 15/150 (10.0%) Anxiolytic: 18/150 (12.0%) Alpha-blocker: 2/150 (1.3%)

LARC, long-acting reversible contraception; OCP, oral contraceptive pill; SD, standard deviation; SNRI, serotonin–norepinephrine reuptake inhibitor; SSRI, selective serotonin reuptake inhibitors.

Most patients (*n* = 137; 91.3%) were taking psychiatric medications at the time of treatment. Of these, most (*n* = 90; 60.0%) were only taking one medication, with 47 (31.3%) engaged in polypharmacy with two (*n* = 39; 26.0%), three (*n* = 5; 3.3%), or four or more (*n* = 3; 2.0%) medications. The most common medication class was SSRIs, with 114 (76%) taking an SSRI, primarily sertraline or fluoxetine, 4 (2.6%) taking a serotonin–norepinephrine reuptake inhibitor (SNRI), 23 (15.3%) taking an atypical antidepressant, 18 (12.0%) taking an atypical antipsychotic, 15 (10%) taking a mood stabilizer, 18 (12.0%) taking an anxiolytic, and 2 (1.3%) taking an alpha blocker. Some patients took more than one medication in each class.

### Patient retention

**FIG. 1.**
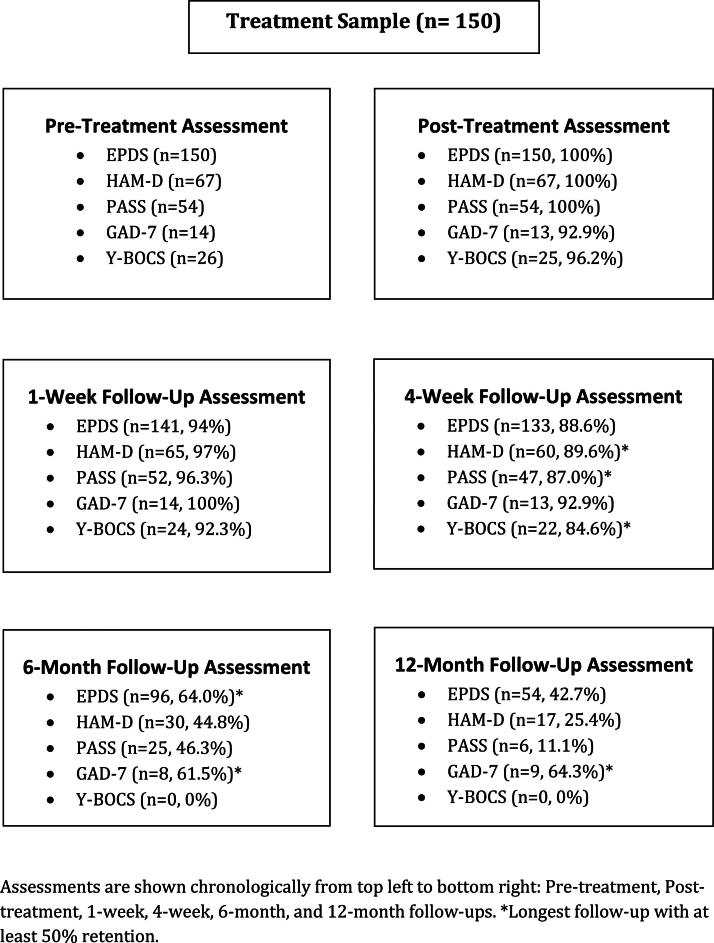
Assessment Completion Rates.

A total of 150 patients underwent brexanolone treatment. Figure 1 includes a participant flow diagram describing assessment rates across time for the different outcome measures. Given the nature of this real-world effectiveness study, rates of retention were highly variable across instruments and time. For inferential analyses using ANOVA, effects were calculated using data from the longest follow-up period with at least 50% follow-up retention, which was 4 weeks for Y-BOC, HAM-D, and PASS, 6 months for the EPDS, and 12 months for the GAD-7. Independent sample *t*-tests comparing patients lost and retained at the above follow-up periods for each measure did not reveal any significant differences. For example, for EPDS, the 96 patients retained at 6 months had similar pretreatment scores (*M* = 19.8, SD = 4.1) to the 54 patients who were not retained (*M* = 20.1, SD = 4.2), *t*(148) = 0.44, *p* = 0.66. None of the other measures were significantly different at pretreatment based on retention. Table 2

**Table 2.** Changes in Outcomes

Rating scale	Pretreatment assessment	Posttreatment assessment	1-Week assessment	4-Week assessment	6-Month assessment	12-Month assessment	*F* (df), *p* *n* Significant pairwise comparisons^a^
EPDS							
Mean (SD)	19.9 (4.1)	10.5 (4.5)	10.8 (5.9)	9.2 (5.6)	9.2 (5.9)	9.2 (6.5)	*F*(3.3, 296.7) = 125.2, *p* < 0.001
*n*	*n* = 150	*n* = 150	*n* = 141	*n* = 133	*n* = 96	*n* = 64	*n* = 90
*d*		*d* = 2.2	*d* = 1.8	*d* = 2.2	*d* = 2.1	*d* = 1.97	SPW: 1, 2, 3, 4, 5
Remission (% <10)	0%	46.7%	47.5%	57.1%	58.3%	60.9%	
Response (≥50%)		68.7%	66.7%	74.4%	75.0%	73.4%	
HAM-D							
Mean (SD)	23.1 (6.2)	12.0 (8.3)	14.3 (7.8)	12.4 (8.4)	11.0 (6.5)	11.5 (7.6)	*F*(3, 177) = 67.1, *p* < 0.001
*n*	*n* = 67	*n* = 67	*n* = 65	*n* = 60	*n* = 30	*n* = 17	*n* = 60
*d*		*d* = 1.5	*d* = 1.2	*d* = 1.4	*d* = 1.9	*d* = 1.7	SPW: 1, 2, 3, 4
Remission (% <8)	0%	37.3%	23.1%	35.0%	36.7%	41.2%	
Response (≥50%)		52.2%	36.9%	55.0%	53.3%	41.2%	
GAD7							
Mean (SD)	14.5 (5.9)	5.1 (4.0)	8.0 (5.0)	8.6 (5.7)	5.0 (2.6)	8.8 (5.7)	*F*(2.0, 4) = 8.3, *p* < 0.01
*n*	*n* = 14	*n* = 13	*n* = 14	*n* = 13	*n* = 8	*n* = 9	*n* = 5
*d*		*d* = 1.9	*d* = 1.2	*d* = 1.0	*d* = 2.1	*d* = 1.0	SPW: none
Remission (% <8)	21.4%	84.6%	35.7%	69.2%	87.5%	44.4%	
Response (≥50%)		92.3%	42.9%	61.5%	87.5%	44.4%	
PASS							*F*(3, 138) = 57.0, *p* < 0.001
Mean (SD)	60.1 (17.3)	33.3 (22.1)	35.4 (20.8)	28.9 (19.9)	27.5 (19.0)	19.7 (29.9)	*n* = 47
*n*	*n* = 54	*n* = 54	*n* = 52	*n* = 47	*n* = 25	*n* = 6	SPW: 1, 2, 3, 4
*d*		*d* = 1.4	*d* = 1.3	*d* = 1.7	*d* = 1.8	*d* = 1.7	
Remission (% <26)	1.9%	48.1%	42.3%	53.2%	48.0%	83.3%	
Response (≥50%)		53.7%	48.1%	61.7%	70.0%	83.3%	
Y-BOCS							
Mean (SD)	18.8 (6.8)	12.4 (7.0)	12.0 (7.4)	10.3 (8.5)	8.6 (6.1)	No data	*F*(3, 54) = 11.9, *p* < 0.001
*n*	*n* = 26	*n* = 25	*n* = 24	*n* = 22	*n* = 8		*n* = 19
*d*		*d* = .9	*d* = 1.0	*d* = 1.1	*d* = 1.5		SPW: 1, 2, 3, 4
Remission (<15)	26.9%	42.3%	54.2%	59.1%	87.5%		
Response (≥35%)		57.6%	62.5%	61.9%	87.5%		

^a^ Pairwise comparisons: 1 = pretreatment to posttreatment, 2 = pretreatment to 1-week, 3 = pretreatment to 4-weeks, 4 = pretreatment to 6-months, 5= pretreatment to 12-months.

EPDS, Edinburgh postnatal depression scale; HAM-D, Hamilton depression rating scale; GAD7, generalized anxiety disorder-7; SD, standard deviation; PASS, perinatal anxiety screening scale; Y-BOCS, Yale–Brown obsessive–compulsive scale.

### Patient safety

There were no REMS reportable adverse events during treatment. However, upon initiation of treatment, early in the dose titration, one patient received a dose exceeding the amount the infusion pump was programmed to deliver due to an infusion set compression spring malfunction. This resulted in the patient receiving the dose programmed to be delivered over 4 hours, in just 1 hour. The patient did not experience an increase in side effects, including sedation, and there was no loss of consciousness. She finished her treatment approximately 3 hours ahead of the scheduled 60-hour treatment. This event was reported to the pump manufacturer through standard reporting channels.

There was one incidental finding during monitored treatment that did not meet the criteria for REMS reporting. A patient with a previous diagnosis of sleep apnea experienced a reduction in pulse oxygen level during sleep, which resolved with elevation of the head of her bed. This incident did not meet the criteria for hypoxia nor necessitate a dosing change or termination of treatment. The patient’s treatment was continued according to the dosing protocol without recurrence.

There were no known serious adverse events during the follow-up period (hospitalizations, suicides, mania, psychosis, serious medical conditions). Approximately 15 patients had a diagnosis of bipolar disorder and were prescribed an antipsychotic or mood stabilizing medication, there was no emergence of mania or hypomania during the follow-up period. During follow-up, one patient self-reported increased fatigue and an increased insulin requirement following treatment with brexanolone that lasted approximately one month following treatment. This was judged unlikely related to brexanolone treatment, and no long-term effects were reported.

### Changes in outcomes

Table 2 includes the means and standard deviations for the entire sample who completed each assessment at each of the included time points, Cohen’s d effect sizes comparing each measurement period to pretreatment, proportions of patients at each time point meeting criteria for remission and response, repeated measures ANOVA significance testing results for changes from pretreatment to the last follow-up period with at least 50% retention, and pairwise comparisons corresponding to the ANOVA.

**FIG. 2.**
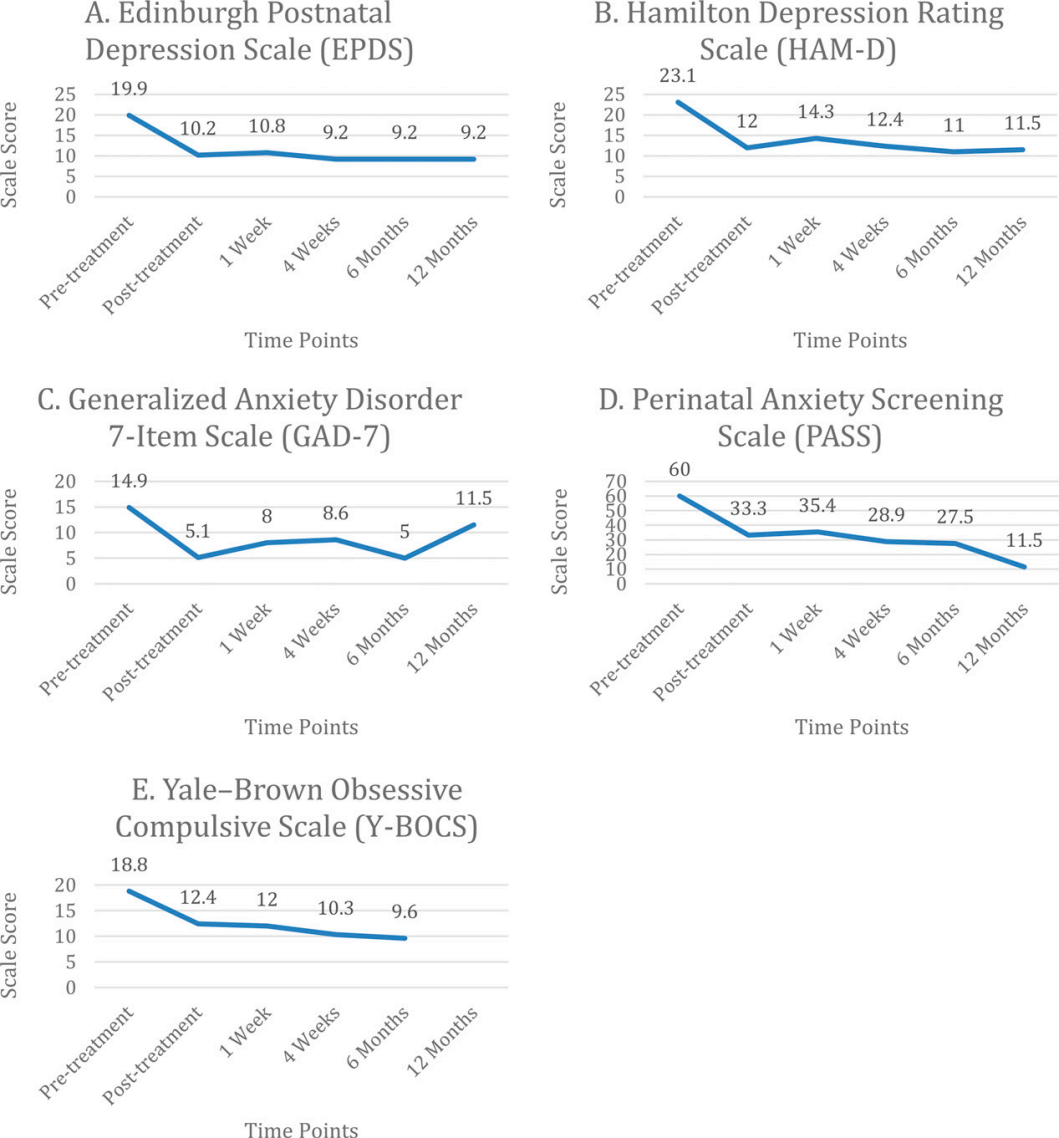
Changes in Outcomes Across Time.

All depression, anxiety, and OCD scores had large changes from pretreatment to posttreatment that sustained across time as measured by Cohen’s d effect sizes. As can be seen in Table 2, these effect sizes ranged from *d* = 0.9 to *d* = 2.2. Additionally, a large proportion of patients achieved remission by dropping below the cutoff score for each of the measures. For example, for the EPDS, rates of remission ranged from 46.7% to 60.9% across the follow-up periods. Repeated measures ANOVA demonstrated a significant time effect with decreases from pretreatment to the latest follow-up for all measures. *Post hoc* pairwise comparisons using Bonferroni correction showed decreased scores between pretreatment and all follow-up assessments (*p* < 0.001) except for GAD-7, which had a very small sample size. Overall, as can be seen in Figure 2, patients' symptoms improved significantly from pretreatment to posttreatment, and these reductions were sustained across the 12-month follow-up period.

## Discussion

### Key insights and novel contributions

Consistent with prior studies, our data show that brexanolone was well tolerated overall and led to statistically significant, large, and rapid within-group effects among community women seeking treatment for PPD. As observed in prior trials, brexanolone had strong antidepressant effects, with an average pre- to posttreatment change of 9.4 points on the EPDS. Nearly, half (46.7%) of patients had an EPDS score in the remission range (<10 at the time of discharge. Adding to the literature, our data demonstrate sustained response and remission in depression symptoms up to 12 months posttreatment, as demonstrated by 73.4% of patients reporting at least a 50% decrease in symptom severity on the EPDS 12 months following treatment.

Our study also adds to the literature by providing insights into the impacts of brexanolone on anxiety. PPD is very often associated with high levels of anxiety, in the form of generalized worry, catastrophic thinking patterns, and intrusive thoughts, which can impact quality of life. While not included as a primary or secondary endpoint in earlier clinical trials, the results of our study demonstrate significant anxiety improvement, with 92.3% of women reporting at least a 50% improvement in symptoms on the GAD-7. Women who screened positive for intrusive thoughts on the EPDS, GAD-7, or PASS were screened for postpartum OCD using the Y-BOCS. While the sample size was small, 87.5% of women experienced at least a 50% decrease in symptoms from pretreatment to 6 months. However, less than half maintained GAD-7 symptom improvement at 12 months, suggesting the impact on anxiety may be less durable or influenced by other confounding factors. Further research may provide additional insights to clarify the role of neurosteroids in treating anxiety disorders during reproductive hormone transition periods.

Our data also expands upon prior trials by including further postpartum patients. Women in our study ranged from 20 to 421 days postpartum (*M* = 127, SD = 88), with 28 patients >6 months postpartum and four >12 months postpartum. Early clinical trials included participants within 6 months postpartum^14,19^ but our findings suggest that treatment can be initiated at any point in the first year. Regular screening for postpartum depression remains crucial throughout the first-year postpartum, including during well-baby visits.

### Limitations

While our real-world data provides compelling effectiveness insights, some limitations require comment. Low follow-up rates at some time points introduce potential bias, though we mitigated this by analyzing only time points with at least 50% retention and checking for baseline differences. Additionally, a high percentage of patients with commercial health insurance and a lack of race and ethnicity data limit generalizability. Lastly, there was no control group, which limits our ability to determine what proportion of improvements observed was attributable specifically to the drug as opposed to the setting, patient expectations, or other factors.

### Applications to zuranolone

The efficacy and safety of brexanolone supported the development of zuranolone, an orally available neuroactive steroid and a positive allosteric modulator of the GABA-A receptor. FDA approved in August 2023, zuranolone demonstrated significant symptom improvement in two large, randomized trials, with effects observed as early as day three and sustained through day 45.^33–35^ The approval of zuranolone reduced treatment burdens, making effective care more accessible. Brexanolone was discontinued as of January 1, 2025. Given their similarities, our findings may be applicable to zuranolone.

### Future directions

PPD remains underrecognized and undertreated, with potentially devastating consequences. Research should continue to enhance PPD treatment development, education, and implementation. Community effectiveness data builds confidence in treatment, increasing the likelihood of screening and patient engagement. Future research should attempt to replicate our findings with zuranolone, focusing on long-term effectiveness and follow-up retention. Hybrid implementation-effectiveness studies may further demonstrate real-world impact and promote adoption among clinicians. Research should also explore factors that moderate treatment effects, such as concurrent antidepressant use, birth control use, breastfeeding status, and psychiatric comorbidities, including insomnia, as well as other demographic or psychosocial factors.

## Conclusion

The last few years have been pivotal in the field of reproductive psychiatry with the approvals of brexanolone and zuranolone. These medications have transformed PPD treatment by providing rapid-acting, short-term, FDA-approved treatments for PPD with the potential for durable relief of depressive symptoms.^34^ Early data suggest their efficacy is comparable with significantly improved response and remission rates over traditional antidepressants.^11,36,37^

Brexanolone represented a true breakthrough in the treatment of PPD and paved the way for zuranolone, which may offer similar benefits with improved accessibility. However, barriers to treatment remain high. Reducing stigma, improving mental health parity, and increasing screening in primary care, pediatric, and obstetric settings is crucial. Identifying and treating PPD is a high-return investment for women, children, families, and communities. We hope for a future where expert mental health care and effective treatment is accessible to all women struggling with reproductive hormone-related mental illness, across the reproductive lifespan.

## Abbreviations Used

ACOG: American College of Obstetricians and Gynecologists
ANOVA: analysis of variance
DSM-5: Diagnostic and Statistical Manual of Mental Disorders, Fifth Edition
EPDS: Edinburgh Postnatal Depression Scale
FDA: U.S. Food and Drug Administration
GABA-A: gamma-aminobutyric acid type A receptor
GAD-7: Generalized Anxiety Disorder 7-item scale
HAM-D: Hamilton Depression Rating Scale
LARC: Long- acting reversible contraceptive
OCD: obsessive–compulsive disorder
OCP: oral contraceptive pill
PASS: Perinatal Anxiety Screening Scale
PHQ-9: Patient Health Questionnaire–9
REMS: Risk Evaluation and Mitigation Strategy
SD: standard deviation
SNRI: serotonin–norepinephrine reuptake inhibitor
SSRI: selective serotonin reuptake inhibitor
Y-BOCS: Yale–Brown Obsessive Compulsive Scale
